# Ultra-high field magnetic resonance imaging of the basal ganglia and related structures

**DOI:** 10.3389/fnhum.2014.00876

**Published:** 2014-11-05

**Authors:** Birgit R. Plantinga, Yasin Temel, Alard Roebroeck, Kâmil Uludağ, Dimo Ivanov, Mark L. Kuijf, Bart M. ter Haar Romenij

**Affiliations:** ^1^Biomedical Image Analysis, Eindhoven University of TechnologyEindhoven, Netherlands; ^2^Department of Neuroscience, Maastricht UniversityMaastricht, Netherlands; ^3^Department of Neurology, Maastricht University Medical CenterMaastricht, Netherlands; ^4^Department of Neurosurgery, Maastricht University Medical CenterMaastricht, Netherlands; ^5^Department of Cognitive Neuroscience, Maastricht UniversityMaastricht, Netherlands; ^6^Department of Biomedical and Information Engineering, Northeastern UniversityShenyang, China

**Keywords:** ultra-high field, magnetic resonance imaging, basal ganglia, thalamus, deep brain stimulation

## Abstract

Deep brain stimulation is a treatment for Parkinson's disease and other related disorders, involving the surgical placement of electrodes in the deeply situated basal ganglia or thalamic structures. Good clinical outcome requires accurate targeting. However, due to limited visibility of the target structures on routine clinical MR images, direct targeting of structures can be challenging. Non-clinical MR scanners with ultra-high magnetic field (7T or higher) have the potential to improve the quality of these images. This technology report provides an overview of the current possibilities of visualizing deep brain stimulation targets and their related structures with the aid of ultra-high field MRI. Reviewed studies showed improved resolution, contrast- and signal-to-noise ratios at ultra-high field. Sequences sensitive to magnetic susceptibility such as T2^*^ and susceptibility weighted imaging and their maps in general showed the best visualization of target structures, including a separation between the subthalamic nucleus and the substantia nigra, the lamina pallidi medialis and lamina pallidi incompleta within the globus pallidus and substructures of the thalamus, including the ventral intermediate nucleus (Vim). This shows that the visibility, identification, and even subdivision of the small deep brain stimulation targets benefit from increased field strength. Although ultra-high field MR imaging is associated with increased risk of geometrical distortions, it has been shown that these distortions can be avoided or corrected to the extent where the effects are limited. The availability of ultra-high field MR scanners for humans seems to provide opportunities for a more accurate targeting for deep brain stimulation in patients with Parkinson's disease and related disorders.

## Introduction

The basal ganglia are a group of nuclei deep in the brain, which play an important role in specific motor, limbic, and associative processes (Temel et al., 2005). Anatomically, they consist of the caudate nucleus-putamen (also referred to as striatum), external and internal globus pallidus (GPe and GPi, respectively), substantia nigra (SN), and the subthalamic nucleus (STN). Structural or functional impairments of basal ganglia structures can lead to neurological and psychiatric disorders, e.g., Parkinson's disease (PD) (Obeso et al., 2008), dystonia (Wichmann and Dostrovsky, 2011), Tourette's syndrome (Mink, 2006), and obsessive-compulsive disorder (Maia et al., 2008). Although most of the patients with basal ganglia diseases can be managed by drug and/or behavioral therapy, an increasing number of patients are referred to specialized teams for deep brain stimulation (DBS) (Lee et al., 2007; Ackermans et al., 2008; Limousin and Martinez-Torres, 2008; Denys et al., 2010). The main reasons for DBS referral include the proven benefit of DBS over best medical treatment (Deuschl et al., 2006; Schuepbach et al., 2013) or insufficient response to non-surgical therapies. DBS is a minimally invasive surgical procedure and involves the implantation of stimulating electrodes with millimeter precision into a specific brain target. The brain regions targeted most often are located in the basal ganglia, and include the ventral parts of the striatum (Malone et al., 2009; Denys et al., 2010), post-eroventral part of the GPi (Damier et al., 2007; Lee et al., 2007; Ackermans et al., 2008), ventral and anterior parts of the pallidum (Ackermans et al., 2008), the STN (Follett and Torres-Russotto, 2012), and surrounding structures such as the ventrolateral and anterior parts of the thalamus (Fisher et al., 2010).

Currently, there are three methods to locate the target for DBS: (a) using intraoperative neurophysiological mapping tools, (b) using stereotactic coordinates derived from *post-mortem* or magnetic resonance imaging (MRI) based atlases (indirect targeting), and (c) via direct visualization on individual magnetic resonance (MR) images (direct targeting). Combinations of these methods are generally used. Direct targeting has the advantage over indirect targeting in that it accounts for differences in individual anatomy, which is especially critical when small structures such as those in DBS are targeted. However, at standard clinical magnetic field strengths (1.5T and 3T) direct visualization often lacks contrast for very high precision DBS targeting. The increasing availability of ultra-high magnetic field (7T or higher) MR scanners promises direct, accurate visualization of target regions with a very high specificity. A better understanding of the structural and functional components of the basal ganglia and related structures at ultra-high resolution approaching the microscopic level, is not only expected to increase the accuracy of DBS, shorten surgery, and potentially improve the clinical outcomes (Yokoyama et al., 2006; Wodarg et al., 2012), but also to enhance our understanding of brain function and disease states. In this technology report, we present the current options for detailed visualization of deep-brain structures using multiple MRI contrasts at ultra-high magnetic field, based on a literature review.

English-language studies were searched on PubMed using combinations of title and abstract key words related to basal ganglia, thalamus, and ultra-high field MRI. Publications were selected by screening of titles and abstracts. Additional studies were found through the references cited in the selected articles.

In this technology report, anatomical structures are denoted in English, unless their Latin names are commonly used. In the first sections, we provide background information on the basic concepts of MRI, which we consider important to understand the different image types that can be obtained, and on the conventional methods of MR imaging of the basal ganglia. Subsequently, we review the current literature on *in vivo* and *ex vivo* (i.e., *post-mortem*) ultra-high field imaging of the basal ganglia and related structures.

## Summary of the principles of magnetic resonance imaging

Whether and how well a certain brain structure is visible on an MR image depends on biophysical tissue parameters and MRI acquisition protocols (see Table 1).

**Table 1:** **Important concepts in MR imaging**.

**Variable**	**Definition**	**Specific for**	**Relevance**
T1	Spin-lattice relaxation time	Tissue	Influences MR signal in tissue
T2	Spin-spin relaxation time		
T2^*^	T2^*^ relaxation time		
R1	1/T1		
R2	1/T2		
R2^*^	1/T2^*^		
TE	Echo time	Sequence	Determines the generated contrast
TR	Repetition time		
Flip angle	Flip angle		
χ	Magnetic susceptibility	Tissue	Gives extra contrast to certain substances
SNR	Signal-to-noise ratio	Image	Quantifies the quality of the image
CNR	Contrast-to-noise ratio		

The relaxation times, T1, T2, and T2^*^, are time constants that describe magnetic spin interaction properties of nuclei, which depend, among other things, on the molecular composition and organization of the tissue and the strength of the main magnetic field. Often, the relaxation rates R1, R2, and R2^*^ are used, defined as 1/T1, 1/T2, and 1/T2^*^ respectively. MRI uses the dependencies of these relaxation times on tissue properties to generate contrast within an image.

The actual type of contrast is determined by the MRI pulse sequence that is used, such as spin-echo (SE) and gradient echo (GE) sequences. These MRI contrasts are sensitive to different biophysical properties of the tissue and it is a matter of intense research to quantitatively relate tissue composition and MRI contrasts. Thus, individual and combinations of MRI contrasts provide a window to examine microstructural properties of brain tissue. The different sequences are described by the combination of the properties of the gradient, radio-frequency pulses and timing parameters. Properties that are often varied are the echo time (TE), repetition time (TR), and flip angle. This can result in T1-, T2- or T2^*^-weighted images in which the contrast is mainly caused by differences in T1, T2, or T2^*^ values of the tissue. The variability in sequences therefore facilitates optimization of the protocol for each structure of interest individually.

### Susceptibility weighted imaging

Susceptibility-weighted (SW) images can also be acquired (Haacke and Reichenbach, 2011). These images are based on the principle that MR images are generally complex-valued, i.e., effectively two images are always acquired: a commonly used magnitude image that often directly displays the anatomical structures and a phase image that is usually disregarded. The phase image however is sensitive to the so-called magnetic susceptibility (χ). This property of tissues and substances alters the local magnetic field values. Paramagnetic materials have a positive χ and strengthen the magnetic field, and diamagnetic materials have a negative χ and weaken the magnetic field. Tissues with a susceptibility that differs from their surrounding structures, such as tissues with myelin and iron-containing substances, cause local deviations in the magnetic field inside and outside of the structures. This leads to local phase differences, which can then be extracted from the original phase images. In susceptibility-weighted imaging (SWI), these phase images are combined with the magnitude images, which can result in additional contrast, which particularly enhances the brain's (micro)vessels and the small deep brain structures.

### Quantitative maps

Furthermore, post-processing techniques can be employed, to produce so-called T1, T2, T2^*^, or (quantitative) susceptibility maps, which display the quantitative T1, T2, T2^*^, or susceptibility values of each voxel in an image respectively. Sometimes R1-, R2- or R2^*^-values are computed instead, which are defined as 1/T1, 1/T2, and 1/T2^*^ respectively.

### Other techniques

In addition to structural imaging, diffusion-weighted imaging (DWI), which is directionally sensitive to water diffusion, gives complementary information (Le Bihan, 2003). It can provide information on the location and orientation of neuronal fibers, aiding in visualization of these pathways (tractography) (Mori et al., 1999) or super-resolution track-density imaging (TDI) (Calamante et al., 2010). Furthermore, functional MRI (fMRI) can provide information on localized brain activity (Buxton, 2013). Finally, DWI and fMRI can be used to compute the connectivity between two areas by computing the fiber paths between them (structural connectivity) or the correlation of functional activity (functional connectivity) respectively.

### Field strength

In most DBS centers, the MR images are obtained from 1.5T or 3T MR scanners. However, in specialized neuroimaging centers, the possibilities of scanning at ultra-high field are increasingly being explored (Duyn, 2012). Although the number keeps growing, at present an estimate of 61 human ultra-high field MR scanners has been installed or will be installed in the near future (see Table 2). At ultra-high field alterations of physical properties can influence measurements both positively and negatively. Several issues including field strength dependent changes in relaxation times T1, T2, and T2^*^; increased B0 and B1 magnetic field inhomogeneities; and increased risks of tissue heating (Duyn, 2012) make ultra-high field scanning more sensitive to inhomogeneous signal-to-noise ratios (SNR) and contrast-to-noise ratios (CNR), geometric distortions, and movement artifacts. This limits the use of T1-weighted, T2-weighted, and proton density weighted turbo spin echo (TSE) scan protocols that are commonly used in clinics (Hennig et al., 1986). However, the alterations in relaxation times and the increased sensitivity to magnetic susceptibility have stimulated the focus of ultra-high field imaging to shift to susceptibility and T2^*^-dependent gradient echo sequences (Haase et al., 2011). Furthermore, the SNR increases close to linearly with field strength, which offers the option to scan with higher spatial resolution (Vaughan et al., 2001) and/or CNR in a shorter time (Duyn, 2012). This makes ultra-high field MRI especially beneficial for detailed imaging of structures with altered magnetic susceptibility, such as the basal ganglia, myelin, and blood, which is also important for ultra-high field high-resolution fMRI imaging. Finally, side effects might occur during movement through the gradients of the strong field. The majority of the subjects have been reported to feel sensations when moving into or out of the bore, which was rated as unpleasant vertigo in 5–20% of the subjects (Glover et al., 2007; Theysohn et al., 2008) and a small number of subjects (approximately 3%) experienced a medium or strong metallic taste (Theysohn et al., 2008).

**Table 2:** **Overview of ultra-high magnetic field (7T or higher) human MR scanners that have been installed or will be installed in the future according to the institutions' websites**.

**Nr**	**Country**	**City**	**Institution, department**	**Manufacturer**	**Field strength (T)**	**Publication**
1	Australia	Melbourne	Melbourne Brain Centre, Melbourne Brain Centre Imaging Unit	Siemens	7	
2	Australia	Brisbane	University of Queensland, Centre for Advanced Imaging	Siemens	7	
3	Austria	Vienna	Medical University of Vienna, MR Center of Excellence	Siemens	7	Hahn et al., 2013
4	Brazil	Sao Paulo	University of Sao Paulo	Siemens	7	
5	Canada	London	Western University, Robarts Research Institute, Centre for Functional and Metabolic Mapping	Siemens	7	Goubran et al., 2014
6	Canada	Toronto	Toronto Western Hospital, Krembil Neuroscience Centre	Siemens	7	
7	China	Beijing	Chinese Academy of Sciences, State Key Laboratory of Brain and Cognitive Science	Siemens	7	He et al., 2014
8	Denmark	Copenhagen	Hvidovre Hospital, Danish Research Centre for Magnetic Resonance	Philips	7	
9	France	Marseille	Center for Magnetic Resonance in Biology and Medicine	Siemens	7	
10	France	Saclay	Alternative Energies and Atomic Energy Commission, Life Sciences Division, Neurospin	Siemens	7	Boulant et al., 2011
11	France	Saclay	Alternative Energies and Atomic Energy Commission, Life Sciences Division, Neurospin	Custom built	11.7	Vedrine et al., 2014
12	Germany	Berlin	Max-Delbrueck-Center for Molecular Medicine, Berlin Ultrahigh Field Facility	Siemens	7	Dieringer et al., 2011
13	Germany	Bonn	German Center for Neurodegenerative Diseases	Siemens	7	
14	Germany	Essen	Erwin L. Hahn Institute for Magnetic Resonance Imaging	Siemens	7	Dammann et al., 2011
15	Germany	Heidelberg	German Cancer Research Center	Siemens	7	Hoffmann et al., 2011
16	Germany	Jülich	Research Centre Jülich, Institute of Neuroscience and Medicine	Siemens	9.4	Arrubla et al., 2013
17	Germany	Leipzig	Max Planck Institute for Human Cognitive and Brain Sciences,	Siemens	7	Deistung et al., 2013a
18	Germany	Magdeburg	Leibniz Institute for Neurobiology, Center for Advanced Imaging	Siemens	7	Hoffmann et al., 2009
19	Germany	Tübingen	Max Planck Institute for Biological Cybernetics	Siemens	9.4	Budde et al., 2014
20	Italy	Pisa	Imago7 Foundation	GE	7	Costagli et al., 2014
21	Japan	Niigata	University of Niigata, Center for Integrated Human Brain Science	GE	7	Kabasawa et al., 2006
22	Japan	Morioka	Iwate Medical University, Institute for Biomedical Sciences	GE	7	Sato and Kawagishi, 2014
23	Japan	Suita City	Center for Information and Neural Networks		7	
24	Netherlands	Leiden	Leiden University Medical Center, C.J. Gorter Center for High Field Magnetic Resonance in the LUMC	Philips	7	Dzyubachyk et al., 2013
25	Netherlands	Utrecht	UMC Utrecht	Philips	7	de Bresser et al., 2013
26	Netherlands	Amsterdam	Spinoza Centre for Neuroimaging	Philips	7	
27	Netherlands	Maastricht	Maastricht University, Maastricht Brain Imaging Centre	Siemens	7	Ivanov et al., 2014
28	Netherlands	Maastricht	Maastricht University, Maastricht Brain Imaging Centre	Siemens	9.4	Cloos et al., 2014
29	Republic of Korea	Icheon	Gachon University of Medicine and Science, Neuroscience Research Institute	Siemens	7	Cho et al., 2008
30	Sweden	Lund	Lund University, Lund University Bioimaging Center	Philips	7	
31	Switzerland	Lausanne	Centre d'Imagerie BioMédicale	Siemens	7	Kickler et al., 2010
32	Switzerland	Zürich	Swiss Federal Institute of Technology and University of Zurich, Institute for Biomedical Engineering	Philips	7	Wyss et al., 2014
33	UK	Nottingham	University of Nottingham, Sir Peter Mansfield Magnetic Resonance Centre	Philips	7	Lotfipour et al., 2012
34	UK	Oxford	University of Oxford, Oxford Centre for Functional Magnetic Resonance Imaging of the Brain	Siemens	7	Berrington et al., 2014
35	USA	Auburn	Auburn University, Magnetic Resonance Imaging Research Center	Siemens	7	Denney et al., 2014
36	USA	Baltimore	Kennedy Krieger Institute, FM Kirby Center for Functional Brain Imaging	Philips	7	Intrapiromkul et al., 2013
37	USA	Bethesda	National Institute of Health, Functional MRI Facility	Siemens	7	Gaitan et al., 2013
38	USA	Bethesda	National Institutes of Health, National Institute of Neurological Disorders and Stroke	Siemens	11.7	
39	USA	Boston	Massachusetts General Hospital, Martinos Center for Biomedical Imaging	Siemens	7	Augustinack et al., 2005
40	USA	Chapel Hill	University of North Carolina		7	
41	USA	Chicago	University of Illinois, Center for MR Research	Custom built	9.4	Lu et al., 2013
42	USA	Cleveland	Cleveland Clinic	Siemens	7	
43	USA	Columbus	Ohio State University, Department of Radiology	Bruker	8	Bourekas et al., 1999; Robitaille et al., 1999
44	USA	Columbus	Ohio State University, Department of Radiology	Philips	7	
45	USA	Minneapolis	University of Minnesota, Center for Magnetic Resonance Research	Siemens	7	Abosch et al., 2010
46	USA	Minneapolis	University of Minnesota, Center for Magnetic Resonance Research	Siemens	7	
47	USA	Minneapolis	University of Minnesota, Center for Magnetic Resonance Research	Siemens	10.5	
48	USA	Minneapolis	University of Minnesota, Center for Magnetic Resonance Research	Varian	9.4	Deelchand et al., 2010
49	USA	Nashville	Vanderbilt University, Institute of Imaging Science	Philips	7	Eapen et al., 2011
50	USA	New Haven	Yale University, Magnetic Resonance Research Center	Varian	7	Pan et al., 2010
51	USA	New York	New York University School of Medicine, Center for Biomedical Imaging	Siemens	7	Pakin et al., 2006
52	USA	New York	Icahn School of Medicine at Mount Sinai, Translational and Molecular Imaging Institute	Siemens	7	
53	USA	Philadelphia	University of Pennsylvania, Center For Magnetic Resonance And Optical Imaging	Siemens	7	Bhagat et al., 2011
54	USA	Pittsburgh	University of Pittsburgh, Magnetic Resonance Research Center	Siemens	7	Moon et al., 2013
55	USA	Portland	Oregon Health & Science University, Advanced Imaging Research Center	Siemens	7	
56	USA	San Francisco	San Francisco Veterans Affairs Medical Center, Center for Imaging of Neurodegenerative Diseases	Siemens	7	
57	USA	San Francisco	University of California, Department of Radiology and Biomedical Imaging	GE	7	Metcalf et al., 2010
58	USA	Dallas	University of Texas Southwestern Medical Center, Advanced Imaging Research Center	Philips	7	Ren et al., 2013
59	USA	Iowa City	University of Iowa, Iowa Institute for Biomedical Imaging	GE	7	
60	USA	Milwaukee	Medical College of Wisconsin, Center for Imaging Research	GE	7	
61	USA	Stanford	Stanford University, Richard M. Lucas Center for Imaging	GE	7	Kerchner et al., 2012

Because not all scanners are operational yet, the last column refers to publications in which the mentioned scanner is used.

## MRI of DBS targets at clinical field strengths of 1.5T and 3T

Direct visualization and targeting of DBS structures based on 1.5T or 3T MR images obtained in clinical practice can be challenging. Several studies compared different scanning sequences for the visibility of the STN (Kerl et al., 2012a; Liu et al., 2013), GPi (Nolte et al., 2012; Liu et al., 2013), GPe (Nolte et al., 2012), and zona incerta (ZI) (Kerl et al., 2012b) and showed that T2^*^ (Kerl et al., 2012a,c; Nolte et al., 2012) and quantitative susceptibility maps (Liu et al., 2013) outperformed T1- and T2-weighted images. Furthermore, 3T functional and structural connectivity maps have been measured in healthy volunteers to visualize the functional subdivision of the STN, although higher spatial resolution is expected to reveal a more detailed anatomy (Brunenberg et al., 2012). Also, a literature review concluded that there is no consensus whether 1.5T and 3T MRI are reliable and accurate enough to be employed for direct targeting of the STN, due to serious shortcomings in the contrast between the STN and surrounding structures (Brunenberg et al., 2011). Visualization of the small substructures in the thalamus at lower field strengths is even less straightforward, primarily due to lack of contrast. One study identified four large thalamic nuclei groups on 3T magnetization-prepared rapid acquisition of gradient echo (MPRAGE) images (Bender et al., 2011) and another study identified the centromedian nucleus directly on 3T proton density weighted MR images (Kanowski et al., 2010). The thalamus was also segmented at 1.5T and 3T using DWI (Wiegell et al., 2003; Unrath et al., 2008; Pouratian et al., 2011; Mang et al., 2012) or a combination of ten different sequences (Yovel and Assaf, 2007).

Although several sequences have been investigated for the visualization of basal ganglia structures at clinical field strengths, DBS structures such as the motor part of the STN, and certain regions within the thalamus, such as the ventrolateral nuclei, need to be displayed more distinctively in order to rely on these images solely for targeting.

## Ultra-high field imaging of the deep-brain structures

Several studies identified deep-brain (sub)structures at ultra-high field using different MRI contrasts. These studies, reviewed below, show the high potential of ultra-high field MRI to accurately identify and delineate thalamic, parathalamic and subthalamic nuclei. Table 3 shows detailed scanning parameters of the described studies, referred to by line numbers.

**Table 3:**
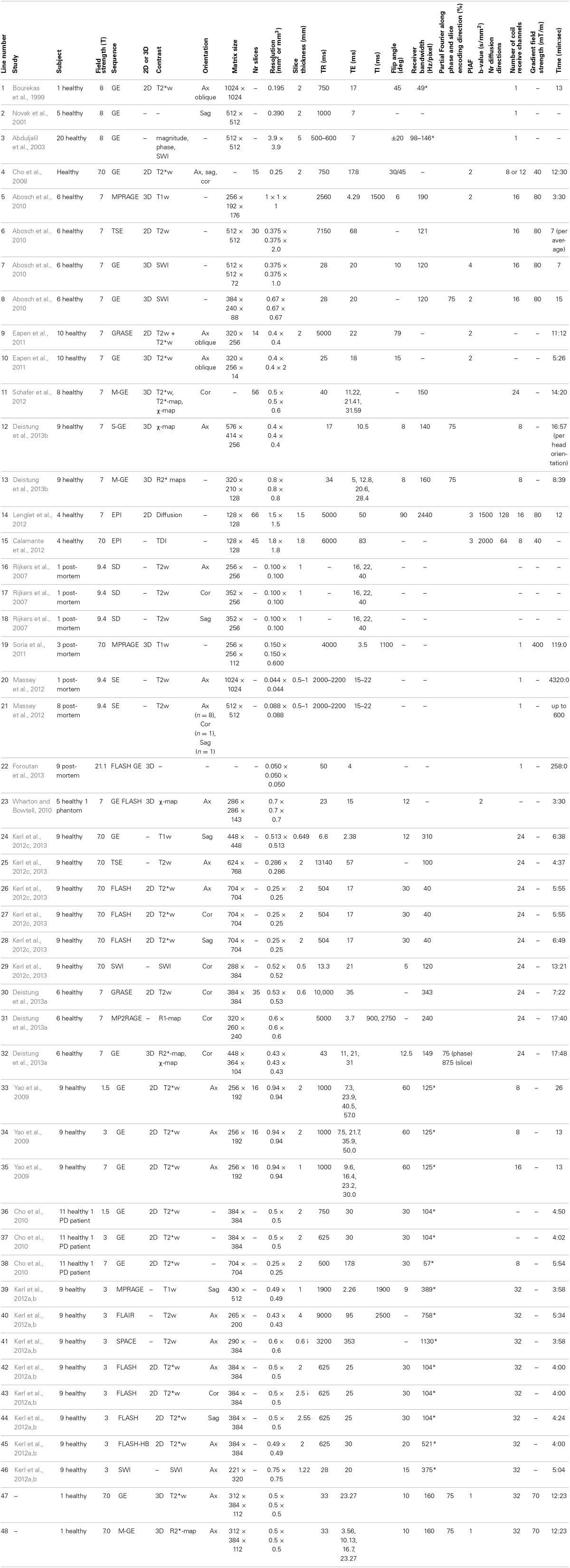
**Overview of acquisition parameters used in the described studies**.

Ax, axial; Cor, coronal; EPI, echo planar imaging; FLAIR, fluid attenuation inversion recovery; FLASH, fast low angle shot; FLASH-HB, FLASH with high bandwidth; FOV, field of view; GE, gradient echo; GRASE, gradient and spin echo; M-GE, multi-echo GE; MPRAGE, magnetization prepared rapid gradient echo; MP2RAGE, magnetization prepared 2 rapid acquisition gradient echoes; PD, Parkinson's disease; PIAF, parallel imaging acceleration factor; Sag, sagittal; SD, spin density; SE, spin-echo; S-GE, single-echo GE; SPACE, sampling perfection with application of optimized contrasts using different flip angle evolutions; SWI, susceptibility weighted imaging; T1w, T1-weighted; T2w, T2-weighted; T2^*^w, T2^*^-weighted; TDI, track density imaging; TI, inversion time; TR, repetition time; TSE, turbo spin-echo; χ-map, susceptibility map. Bandwidths that were originally reported in kHz have been converted to Hz/pixel and are denoted with an asterisk (^*^).

### Visualization of deep-brain structures at ultra-high field *in vivo*

Since the installation of the first ultra-high field MR scanner, several studies investigated the visualization of deep-brain structures at ultra-high field *in vivo* (Table 4).

**Table 4:** **Overview of the basal ganglia and related (sub)structures that have been identified using different protocols at ultra-high field MRI**.

**Study**	**Image type**	**Findings**	**Line**
Bourekas et al., 1999	T2^*^w	GP, SN, and RN appear hypointense	1
Novak et al., 2001		GP, SN, and RN appear hypointense	2
Abduljalil et al., 2003	GE Magnitude	SN and RN appear hypointense	3
	GE Phase	Substructures within SN and RN	3
Cho et al., 2008	GE	SN and RN in coronal plane hypointense	4
Cho et al., 2010	Coronal GE	Discrimination of STN and SN	38
Abosch et al., 2010	SWI	Clear delineation of STN	7–8
		Boundary between STN and SN	
		Lamina pallidi medialis and lamina pallidi incompleta	
		Vim, anterior and medial boundaries of pulvinar, boundary of the nucleus ventralis caudalis	
Eapen et al., 2011	T2w and T2^*^w	Subregions within RN	9
	T2^*^w	Subregions within RN and SN	10
Schafer et al., 2012	χ-map	Boundary between STN and SN	11
Deistung et al., 2013b	χ-map	Subnuclei within the SN	12
		Discrimination of the STN from the SN and surrounding gray and white matter	
		Lamina pallidi medialis and lamina pallidi incompleta	
		Medullary lamina in RN	
		Vim, pulvinar, lateral and medial geniculate nucleus, dorsomedial nucleus and dorsal nuclei group	
	R2^*^-map	Substructures in RN	13
Lenglet et al., 2012	Tractography	Projection based subdivisions of the SN, STN, GP and thalamus	14
Calamante et al., 2012	TDI	Signal intensity differences within thalamus	15
***POST-MORTEM* STUDIES**			
Rijkers et al., 2007	T2w	Visualization of the pulvinar, the lateral and medial geniculate bodies, cerebral peduncle, habenulointerpeduncular tract, periaquaductal gray, the medial lemniscus, the spinothalamic tract, the mammillothalamic tract, and the superior colliculus.	16:18
Soria et al., 2011	T1w	Visibility of SN and RN	19
Massey et al., 2012	T2w	Hypointense band between SN and STN	21
		High detailed visibility of STN and surrounding	
		Intensity differences between anteromedial and posterolateral part of STN	
	T2w	Fibers of the subthalamic fasciculus	20
Foroutan et al., 2013	FLASH GE	High-detail images of SN, RN, putamen, and a clear separation of the GP into its external and internal part.	22

The last column refers to the line of Table 3 that gives more details about the scan protocols used. FLASH, fast low angle shot; GE, gradient echo; GP, globus pallidus; RN, red nucleus; SN, substantia nigra; STN, subthalamic nucleus; SWI, susceptibility-weighted imaging; T1w, T1-weighted; T2w, T2-weighted; T2^*^w, T2^*^-weighted; TDI, track density imaging; Vim, ventral intermediate nucleus; χ-map, susceptibility map.

In 1999, the basal ganglia were visualized at ultra-high field (8T) using a two-dimensional (2D) multi-slice GE sequence, where high-resolution (195 × 195 μm in-plane) T2^*^-weighted axial images of one volunteer were obtained in 13 min (Table 3-1) (Bourekas et al., 1999). On these images the globus pallidus (GP), SN and red nucleus (RN) appeared as hypointense regions. These findings were later confirmed in sagittally recorded slices with similar acquisition parameters (Table 3-2) (Novak et al., 2001). In 2003, the same group showed that on GE phase images (Table 3-3), within the SN, the SN pars dorsalis and SN pars lateralis had a higher signal intensity than the matrix of the SN, and within the RN, the medullary lamella showed a higher signal intensity than the RN pars oralis (Abduljalil et al., 2003). A few years later, again the SN and RN appeared hypointense on 7T axial, sagittal, and coronal GE images (Table 3-4) (Cho et al., 2008) and in 2010, 7T coronal GE images (Table 3-38) were obtained on which the STN and SN could be well distinguished (Cho et al., 2010).

**Figure 1:**
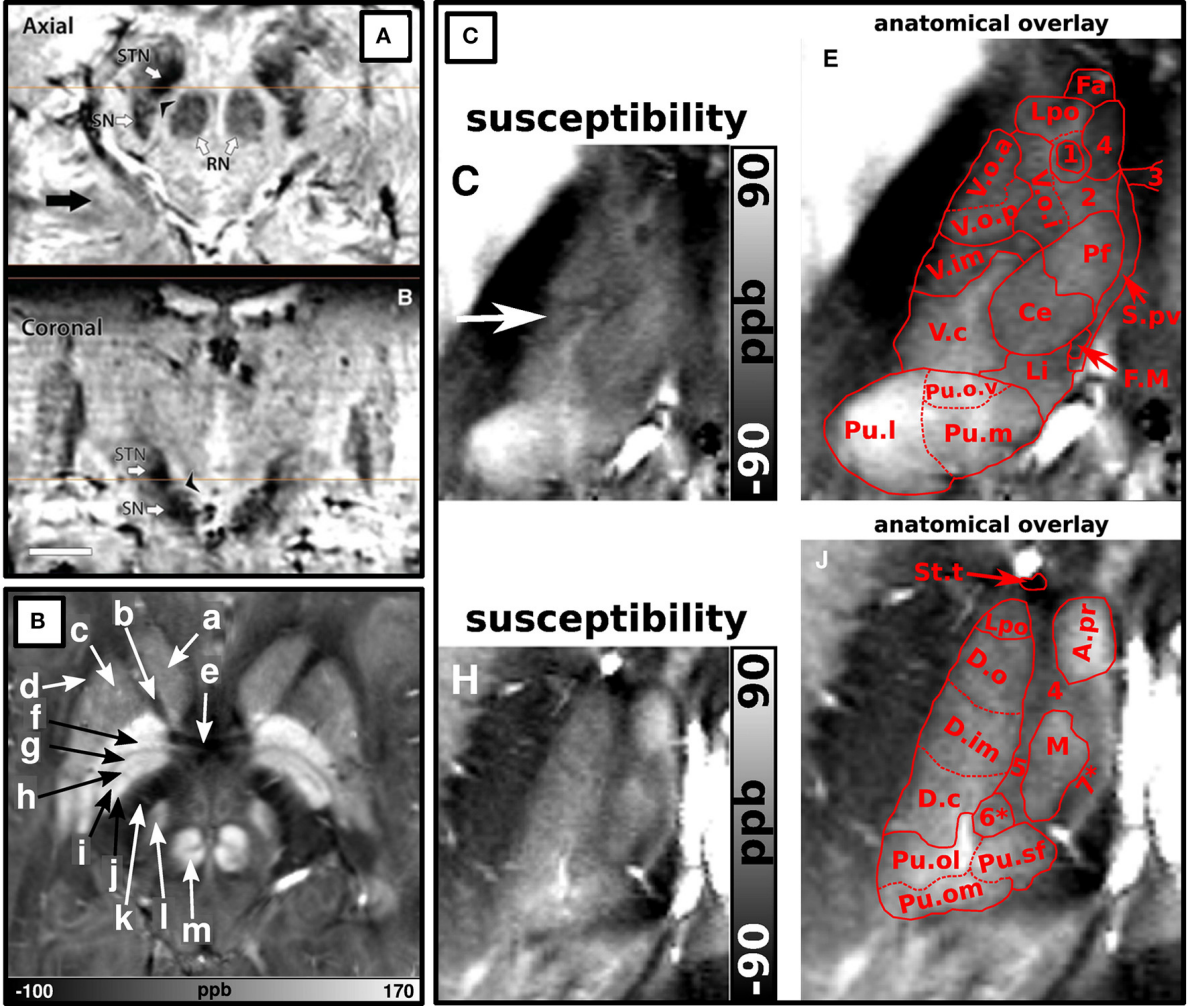
**Examples of structures identified at ultra-high field. (A)** Adopted with permission from Abosch et al. (2010). Ultra-high field (7T) susceptibility-weighted axial and coronal images show a clearly delineated subthalamic nucleus (STN), a boundary between the STN and substantia nigra, and heterogeneous signal intensity in the red nucleus. **(B)** Adopted with permission from Deistung et al. (2013b). Axial 7T susceptibility map displaying (a) the head of the caudate nucleus, (b) anterior limb of the internal capsule, (c) putamen, (d) external capsule, (e) anterior commissure, (f) external globus pallidus, (g) lamina pallidi medialis, (h) pallidum mediale externum, (i) lamina pallidi incompleta, (j) pallidum mediale internum, (k) posterior limb of internal capsule, (l) subthalamic nucleus, and (m) red nucleus. **(C)** Adopted with permission from Deistung et al. (2013b). Ultra-high field (7T) susceptibility maps of inferior (C,E) and superior (H,J) sections of the thalamus. (E,J) show overlays of substructures of the thalamus according to the Schaltenbrand et al. (1977) on the images shown in (C,H) respectively. The pulvinar (Pu.l) can be distinguished from (C,E) and the dorsomedial nucleus (M) and dorsal nuclei group (D.o and D.im) can be seen in (H,J).

A more detailed description of the visualization of the basal ganglia at 7T with three different scanning sequences, exploiting T1-weighted, T2-weighted and susceptibility-weighted imaging, was published in 2010 (Table 3-5:8) (Abosch et al., 2010). Using SWI, a clear delineation of the STN and the boundary dividing it from the SN were visualized in both axial and coronal planes (Figure 1A). Also, SWI allowed visualization of varying levels of contrast within the RN and two of the laminae within the GP (lamina pallidi medialis and incompleta), thus also distinguishing between the GPi and the GPe. Within the thalamus, it showed intensity variations corresponding to the locations of the ventral intermediate nucleus (Vim), the anterior and medial boundaries of the pulvinar, and the boundary of the nucleus ventralis caudalis as identified with the Schaltenbrand and Wahren atlas (Schaltenbrand et al., 1977).

In 2011, Eapen et al., imaged several deep-brain structures with two different sequences at 7T: T2- and T2^*^-weighted gradient and spin-echo (GRASE) and T2^*^-weighted GE (Table 3-9:10) (Eapen et al., 2011). Both GRASE and GE scans showed a clear distinction between the densely and the poorly vascularized regions of the RN, but only the GE scan also showed signal intensity differences within the SN, possibly representing the SN pars compacta and SN pars reticulata. In two later studies, susceptibility maps were investigated. Using a multi-echo GE sequence (Table 3-11), a boundary between the STN and the SN was shown (Schafer et al., 2012). The use of susceptibility maps generated from three single-echo GE phase data sets with different head positions (Table 3-12) also facilitated detailed visualization of structures (Deistung et al., 2013b). It provided discrimination between the subnuclei within the SN, and allowed for accurate discrimination of the STN from the SN and surrounding gray matter and white matter. Furthermore, within the GP, these maps showed the lamina pallidi medialis and lamina pallidi incompleta (Figure 1B). The RN displayed substructures in the susceptibility maps, facilitating identification of the medullary lamella, and the RN pars oralis and RN pars dorsomedialis showed a significantly increased susceptibility, compared to the RN pars caudalis. Finally, within the thalamus clear intensity variations were observed on these susceptibility maps corresponding to the Vim, pulvinar, lateral and medial geniculate nucleus, dorsomedial nucleus, and dorsal nuclei group as identified with the Schaltenbrand and Wahren atlas (Schaltenbrand et al., 1977) (Figure 1C).

In two other studies by Kerl et al., investigating the STN and ZI with different sequences at 7T (Kerl et al., 2012c, 2013), a distinction between the STN and the SN and ZI and a clear boundary dividing the rostral ZI from the internal capsule, STN and the pallidofugal fibers could be seen on T2^*^-weighted images and the latter also on coronal SW images.

Finally, two studies employed DWI properties to identify substructures within the DBS related structures. In one study, DWI (Table 3-14) was used to estimate the pathways between seven regions of interest: caudate nucleus, putamen, GPe, GPi, SN, STN, and thalamus (Lenglet et al., 2012). Seven pathways could be successfully identified: the nigrostriatal, nigropallidal, nigrothalamic, subthalamopallidal, pallidothalamic, striatopallidal, and thalamostriatal pathway. These projections were also used to create subparcellations of the SN, possibly corresponding to the SN pars reticulata and SN pars compacta; subdivisions of the STN into a dorsolateral and ventromedial part; subdivisions of the GPe into medial, lateral and rostro-ventral parts; subdivisions of the GPi into laterocaudal, rostral, and mid portions; and many subdivisions within the thalamus. In another study, 7T DWI (Table 3-15) was used to construct track-density images of the thalamus (Calamante et al., 2012). These showed high-resolution (200 μm isotropic) substructures within the thalamus with clear intensity differences, not only related to track-density, but also to the directionality of the fibers.

### Visualization of deep-brain structures at ultra-high field *ex vivo*

When scanning *ex vivo*, even higher resolution and higher SNR can be obtained due to the possibility of longer scan times and less movement artifacts. Although fixed tissue may suffer from altered tissue properties, such as decreases in T1 and T2 (Tovi and Ericsson, 1992) and a decreased diffusion coefficient (D'Arceuil et al., 2007), which is especially challenging for DWI, it also has great advantages over *in vivo* MRI. Several studies employed *ex-vivo* imaging for investigating the deep-brain structures at ultra-high field (Table 4).

In 2007, the STN and its surroundings were explored at 9.4T with a T2-weighted sequence (Table 3-16:18) in a *post-mortem* brain sample (Rijkers et al., 2007). Acquiring a high in-plane resolution of 100 × 100 μm, not only the most prominent structures of the basal ganglia were visualized, but also the pulvinar, the lateral and medial geniculate bodies, cerebral peduncle, habenulointerpeduncular tract (fasciculus retroflexus), periaquaductal gray, the medial lemniscus, the spinothalamic tract, the mammillothalamic tract, and the superior colliculus.

Three *post-mortem* brain stems have also been imaged at 7T for 119 min, acquiring 150 × 150 μm images. On these T1-weighted images (Table 3-19), the RN and SN, which displayed heterogeneous signal intensity, could be visualized (Soria et al., 2011). Even higher in-plane resolutions of 44 × 44 and 88 × 88 μm (Table 3-20:21) were achieved in a different study after scanning *post-mortem* brain samples for 72 and 10 h respectively (Massey et al., 2012). The obtained T2-weighted images facilitated visualization of the STN, SN, RN, ZI, and thalamus but also allowed a highly detailed identification of many smaller structures surrounding the STN. Furthermore, a hypointense signal band was seen between the SN and STN facilitating easy separation of the two structures. Also the anteromedial part of the STN was relatively hypointense compared to the posterolateral portion, which might be related to the subdivision of the STN in a limbic, associative and sensorimotor part. On the 44 × 44 μm resolution images even the fibers of the subthalamic fasciculus were visualized accurately.

Finally, one study that focused on differences in T2 and T2^*^ values and iron content between *post-mortem* brains of progressive supranuclear palsy patients and controls, showed high-resolution (50 μm isotropic) fast low-angle shot (FLASH) GE images (Table 3-22), displaying with much detail the SN, RN, putamen, and the GP with a clear separation into the GPe and GPi (Foroutan et al., 2013).

These studies show that ultra-high field MRI can aid substantially in the identification of small (sub)structures including the separation between the STN and SN and the laminae within the GP both *ex vivo* and *in vivo*.

### Comparison between sequences for ultra-high field imaging

In addition to the qualitative description of the visibility of deep-brain structures with ultra-high field MRI, comparisons between different sequences and image reconstruction methods have been made (see Table 5).

**Table 5:** **Overview of comparative studies at ultra-high field**.

**Study**	**Sequences**	**Line**	**Measure**	**Findings**
Abduljalil et al., 2003	GE magnitude	3	Qualitative	Phase images show additional structures to magnitude images
	GE SWI	3		
	GE phase	3		Magnitude + Phase ≥ SWI
Wharton and Bowtell, 2010	MO χ-map	23	Artifacts and Δχ	Least noise related artifact and most accurate Δχ in MO
	RSO χ-map	23		
	TSO χ-map	23		MO≈RSO≈TSO
Abosch et al., 2010	T1w	5	Qualitative	SWI > T2w > T1w
	T2w	6		
	SWI	7:8		
Eapen et al., 2011	T2w + T2^*^w	9	CNR of RN/VTA	T2w + T2^*^w > T2^*^w
	T2^*^w	10		
Schafer et al., 2012	T2^*^w	11	CNR	χ-map > T2^*^w > T2^*^-map
	T2^*^-maps	11		
	χ-map	11		
Kerl et al., 2012c, 2013	T1w	24	SNR STN	T2^*^w^‡^ > T1w^‡^ > SWI-MIP^‡^ > SWI cor^‡^ > T2w
	T2w	25	CNR STN	T2^*^w^‡^ > SWI-MIP^‡^ > T2 > SWI > T1w
	T2^*^w	26:28	SNR rZI	T2^*^w^‡^ > SWI-MIP^‡^>T1^‡^>SWI>T2w
	SWI	29	CNR rZI	T2^*^w^‡^ > SWI-MIP^‡^ >T2>SWI>T1w
	SWI-MIP	29		
Deistung et al., 2013b	GE magnitude	12	Qualitative	χ-map showed most detail
	GE phase	12		
	χ-map	12		
	R2^*^-map	13		
Deistung et al., 2013a	T2w	30	CNR SN	χ-map > R2^*^-map > T2w > R1-map
	R1-map	31	CNR RN	χ-map > R2^*^-map > T2w > R1-map
	R2^*^-map	32		
	χ-map	32		

The third column refers to the line of Table 3 that gives more details about the scan protocols used. Sequences that give significantly better results than T2-weighted images are denoted with a double dagger (^‡^). CNR, contrast-to-noise ratio; cor, coronal; GE, gradient echo; MIP, minimum intensity projection; MO, multi-orientation; RN, red nucleus; RSO, regularized single-orientation; rZI, rostral part of zona incerta; SNR, signal-to-noise ratio; STN, subthalamic nucleus; SWI, susceptibility-weighted imaging; T1w, T1-weighted; T2w, T2-weighted; T2^*^w, T2^*^-weighted; TSO, threshold based single orientation; VTA, ventral tegmental area; χ-map, susceptibility map.

In a previously mentioned study from 2003, magnitude, phase-weighted magnitude (SWI), and phase images of a GE dataset (Table 3-3), were compared for their capability to visualize (sub)structures (Abduljalil et al., 2003). On magnitude images the SN and RN showed up hypointense and on phase images, substructures within the SN could be distinguished as well. The combined magnitude and phase images added little extra to the magnitude and phase images separately.

Later, in 2010, the mean susceptibility difference (Δχ) between compartments in an agar phantom, and between white matter and deep-brain structures of healthy subjects were compared among three different susceptibility mapping methods applied to GE FLASH images acquired at 7T (Table 3-23) (Wharton and Bowtell, 2010). The mapping methods consisted of (a) a multi-orientation method using images acquired with differing head positions, (b) a regularized single-orientation method, and (c) a threshold-based single-orientation method. Although all three methods showed large Δχ in the GP, SN, RN, internal capsule, putamen and caudate nucleus, the multi-orientation method resulted in the least noise related artifacts and good estimation of Δχ values in the phantom.

**Figure 2:**
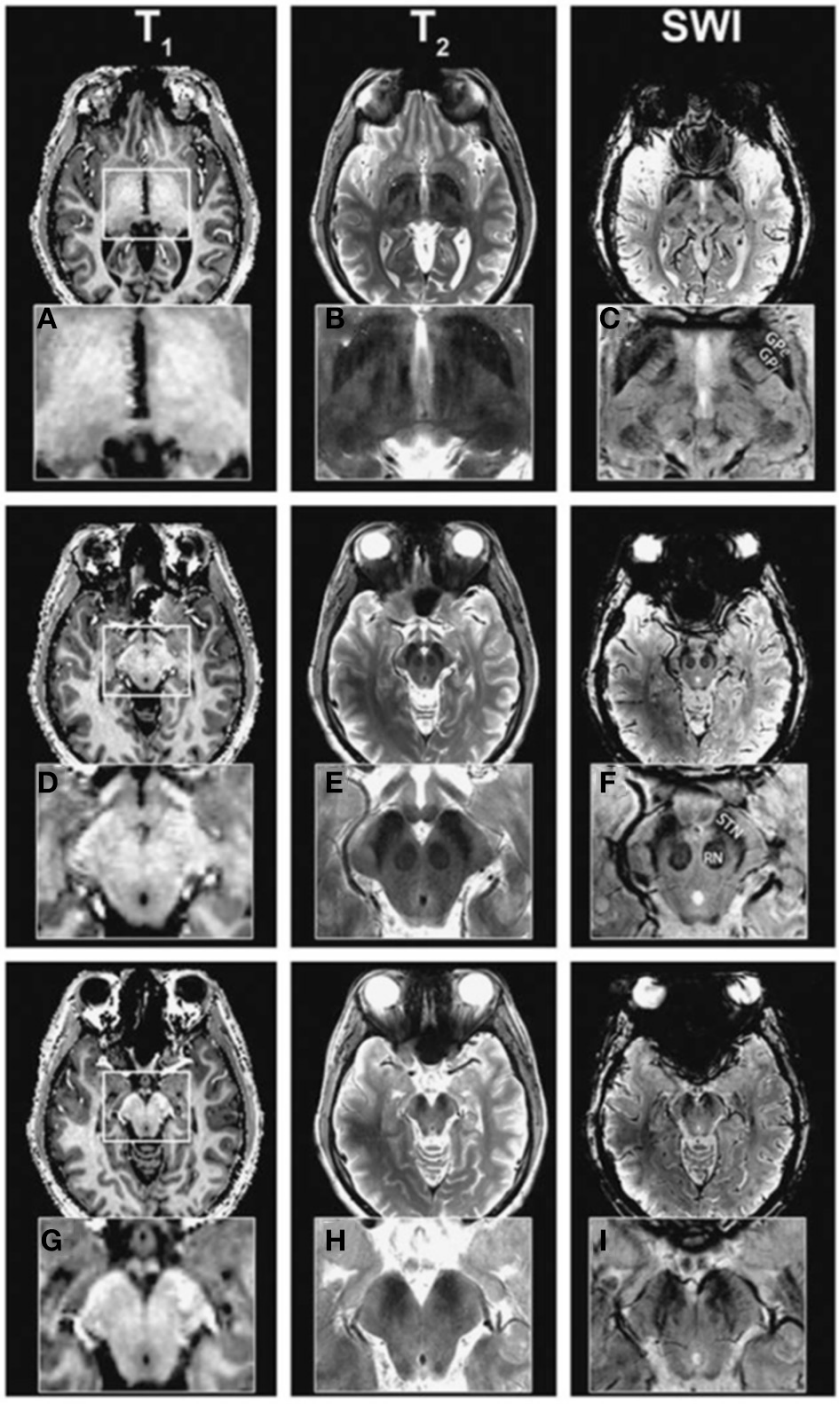
**Ultra-high field (7T) T1-weighted (A,D,G), T2-weighted (B,E,H), and susceptibility-weighted (C,F,I) images at different levels.** Adopted with permission from Abosch et al. (2010). The susceptibility-weighted images show the highest detail followed by the T2-weighted images.

In another 2010 study, T1-weighted, T2-weighted, and SW imaging (Table 3-5:8) were compared (Abosch et al., 2010). Most structures were identified in the SW images (see Table 4), followed by the T2-weighted images (Figure 2). The T1-weighted images showed no obvious structures. Eapen et al., also quantitatively compared their T2 + T2^*^- and T2^*^-weighted images (Table 3-9:10) (Eapen et al., 2011). No difference between both sequences could be found in the CNR between the SN and ventral tegmental area (VTA) and between the SN and RN, but in the T2 + T2^*^-weighted images, the CNR between RN and VTA was significantly better than in the T2^*^-weighted images.

In 2012, again differently reconstructed images derived from a multi-echo GE sequence (Table 3-11) were compared, consisting of T2^*^-weighted magnitude images, T2^*^-maps, and susceptibility maps (Schafer et al., 2012). In most subjects, the CNR between the SN and STN was highest in the susceptibility maps, suggesting that these are most suitable for differentiating the STN from the SN. The SNR of the STN and the rostral part of the ZI (rZI) and the CNR between these structures and white matter, imaged with different sequences, were investigated in two recent studies that compared T1-weighted GE, T2-weighted TSE, T2^*^-weighted FLASH and SW images (Table 3-24:29) (Kerl et al., 2012c, 2013). Furthermore, minimum intensity projections (MIPs) of the SW images were computed. After adjusting the SNR and CNR for differences in voxel size, they were highest on the T2^*^-weighted images for both structures. Furthermore, the SNRs of both structures on the T2^*^-weighted, T1-weighted, SWI-MIP (and for the STN also on the coronal SW images) were significantly higher than those of the T2-weighted images. The CNRs of both structures on the T2^*^-weighted and for the rZI also on the SWI-MIP images were also significantly higher than on the T2-weighted images. Also, a 2013 study compared image reconstruction techniques at 7T consisting of (a) magnitude, (b) frequency, and (c) susceptibility maps derived from GE scans (Table 3-12), and (d) R^*^_2_ maps derived from multi-echo GE scans (Table 3-13) (Deistung et al., 2013b). Qualitative analysis by a neuroanatomist revealed that susceptibility maps in general facilitated the most detailed visualization of structures. Finally, in a recent study by the same group, the CNR between several brain stem structures and their surroundings were compared between sequences (Table 3-30:32) (Deistung et al., 2013a). For the RN and the SN, the CNR of the R2^*^-map and the susceptibility map outperformed those of the R1-map and the T2-weighted image.

Although comparison between studies is difficult due to the differences in scanning conditions, the majority of these studies show that sequences that are sensitive to magnetic susceptibility such as SWI and T2^*^ related images are most suitable for targeting basal ganglia structures and their subdivisions in DBS at ultra-high field.

### Comparison between field strengths

**Figure 3:**
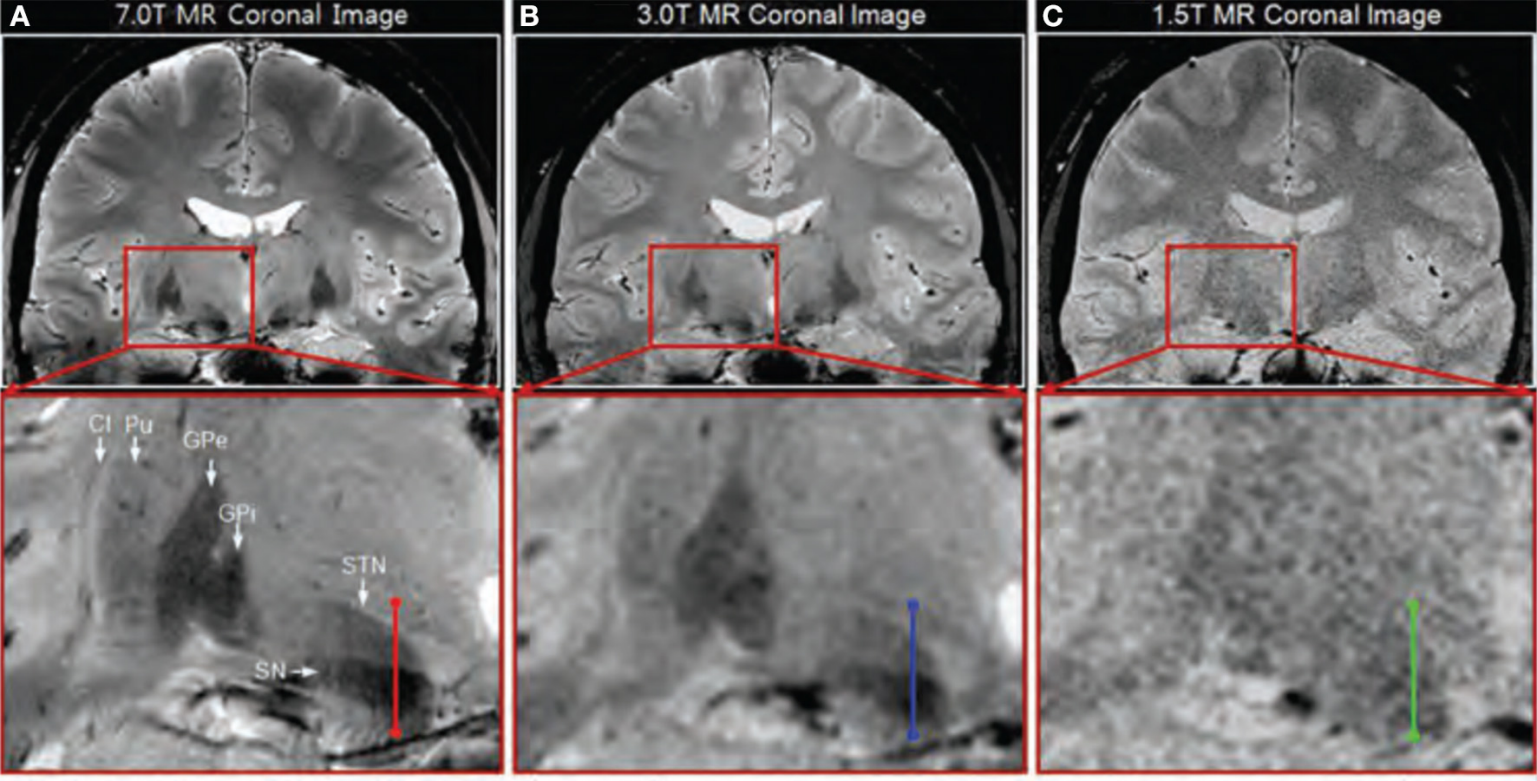
Coronal T2^*^-weighted images obtained at 7.0T (A), 3.0T (B), and 1.5T (C). Adapted with permission from Cho et al. (2010). Visual inspection shows clearer identification of the substantia nigra (SN), subthalamic nucleus (STN), internal globus pallidus (GPi), external globus pallidus (GPe), and putamen (Pu) at 7T compared to 3T and 1.5T.

In addition to comparisons between different sequences, some studies compared similar sequences between different field strengths (see Table 6). In a 2008 study, the difference between a 7T GE image (Table 3-4) and a 1.5 T image was briefly treated (Cho et al., 2008). Visual inspection showed that the 7T image displayed better contrast, SNR and resolution. However, comparison is difficult because the acquisition parameters of the 1.5T image were unfortunately not provided. In the same year, T2^*^-weighted GE images were investigated, acquired at several echo times at three different field strengths: 1.5T, 3T, and 7T (Table 3-33:35) (Yao et al., 2009). This showed that increasing field strength resulted in a higher influence of iron on the value of R2^*^, making this contrast useful for iron-rich deep-brain structures, such as the GP, RN, SN, and putamen (Hallgren and Sourander, 1958). A thorough quantitative investigation of the visibility of the STN related to field strength was performed in 2010 (Cho et al., 2010), comparing the contrast between the STN and a baseline (containing the ZI and thalamus), the contrast between the STN and SN, the SNR in gray matter areas, and the slope of signal increase between STN and baseline among 1.5T, 3T, and 7T T2^*^-weighted GE images (Table 3-36:38). At higher field strengths, the STN, SN, putamen, GPi, and GPe could be visualized while the boundaries of these structures were unclear on the 1.5T images (Figure 3). Furthermore, all quantitative measures increased with field strength, and the SNR and contrast were significantly improved at 7T compared to 1.5 and 3T. Finally, the two studies by Kerl et al., investigating the STN and rZI at 7T (Kerl et al., 2012c, 2013) were additionally performed at 3T. Again, they compared the SNR and CNR of these structures between different sequences: T1-weighted MPRAGE, T2-weighted fluid attenuated inversion recovery (FLAIR), T2-weighted sampling perfection with application of optimized contrasts using different flip angle evolutions (SPACE), two T2^*^-weighted 2D FLASH (FLASH2D) sequences, and SW images and their MIPs (Table 3-39:46) (Kerl et al., 2012a,b). This makes it possible to compare the SNRs and CNRs of the different studies between field strengths, when adjusted for voxel size, although it should be noted that for the T1- and T2-weighted images different sequences were used between field strengths. For both structures, the SNRs of the T2^*^-weighted, SWI-MIP and SW images of the 7T images were higher than those of the 3T images, but the SNRs of the 3T T2-weighted SPACE image and T1-weighted images were higher at 3T than at 7T. However, the CNRs of both structures were substantially higher on all the 7T sequences than on the corresponding 3T sequences.

**Table 6:** **Overview of studies that compare scan protocols between field strengths**.

**Study**	**Sequence**	**Line**	**Measure**	**Findings**
Cho et al., 2008	1. 1.5T	4	Qualitative	7T has better contrast, SNR and resolution than 1.5T
	2. 7T T2^*^w			
Yao et al., 2009	1. 1.5T T2^*^w	33	R2^*^	R2^*^ becomes more sensitive to iron with increasing field strength
	2. 3T T2^*^w	34		
	3. 7T T2^*^w	35		
Cho et al., 2010	1. 1.5T T2^*^w	36	Contrast	7T^‡^>3T>1.5T
	2. 3T T2^*^w	37	Slope of signal increase	7T>3T>1.5T
	3. 7T T2^*^w	38	SNR	7T^‡^>3T>1.5T
Kerl et al., 2012a,b,c, 2013	1. 3T T1w	39	SNR	3T T1w > 7T T1w
	2. 3T T2w FLAIR	40		7T T2^*^w > 3T T2^*^w
	3. 3T T2w SPACE	41		7T SWI-MIP > 3T SWI-MIP axial
	4. 3T T2^*^w	41:45		3T T2w SPACE > 7T T2w > 3T T2w FLAIR
	5. 3T SWI	46		7T SWI > 3T SWI
	6. 3T SWI-MIP	46		
	7. 7T T1w	24	CNR	7T T2^*^w > 3T T2^*^w
	8. 7T T2w TSE	25		7T SWI-MIP > 3T SWI-MIP
	9. 7T T2^*^w	26:28		7T T2w > 3T T2w SPACE > 3T T2w FLAIR
	10. 7T SWI	29		7T SWI > 3T SWI
	11. 7T SWI-MIP	29		7T T1 > 3T T1

The third column refers to the line of Table 3 that gives more details about the scan protocols used. Sequences that significantly improve imaging at 7T compared to 1.5T and 3T are denoted with a double dagger (‡). CNR, contrast-to-noise ratio; FLAIR, fluid attenuated inversion recovery; MIP, minimum intensity projection; rZI, rostral part of zona incerta; SNR, signal-to-noise ratio; SPACE, sampling perfection with application of optimized contrasts using different flip angle evolutions; STN, subthalamic nucleus; SWI, susceptibility-weighted imaging; T1w, T1-weighted; T2w, T2-weighted; T2^*^w, T2^*^-weighted; TSE, turbo spin-echo.

These studies suggest that 7T MRI can better facilitate accurate targeting of deep brain structures than 1.5T or 3T MRI.

## Discussion

Accurate visualization of deep-brain structures is important to improve our understanding of their anatomy, connectivity and function, and for improved surgical targeting for DBS in movement and psychiatric disorders. To date, targeting based on direct visualization of DBS targets with T2-weighted 1.5T or 3T MRI can be difficult. However, studies at ultra-high field showed good visibility of these structures on SW images based on T2^*^ and phase contrast. Structures that have been identified at ultra-high field include: a separation between the STN and SN (Abosch et al., 2010; Cho et al., 2010; Massey et al., 2012; Schafer et al., 2012; Deistung et al., 2013b); the lamina pallidi medialis and lamina pallidi incompleta within the GP (Abosch et al., 2010; Deistung et al., 2013b); a subdivision of the STN in two halves (Lenglet et al., 2012; Massey et al., 2012); subdivisions of the SN possibly representing the SN pars reticulata and SN pars compacta (Eapen et al., 2011; Lenglet et al., 2012; Deistung et al., 2013b); substructures in the RN (Abosch et al., 2010; Eapen et al., 2011) including the medullary lamella (Abduljalil et al., 2003; Deistung et al., 2013b), RN pars oralis (Abduljalil et al., 2003), and RN pars caudalis (Deistung et al., 2013b); and several regions in the thalamus (Lenglet et al., 2012) including the Vim (Abosch et al., 2010; Deistung et al., 2013b), the pulvinar (Deistung et al., 2013b) and its anterior and medial boundaries (Abosch et al., 2010), the boundary of the nucleus ventralis caudalis (Abosch et al., 2010), the lateral and medial geniculate nucleus (Deistung et al., 2013b), the dorsomedial nucleus (Deistung et al., 2013b) and the dorsal nuclei group (Deistung et al., 2013b). Furthermore, 7T T2^*^-weighted and SW images have displayed improved CNR, SNR and resolution in the deep-brain regions, compared to 1.5T and 3T images (Cho et al., 2010; Kerl et al., 2012a,b,c, 2013).

Based on a descriptive evaluation of different MR images, more and smaller structures can be identified on T2^*^-weighted, GE phase, SW images, and susceptibility and R2^*^ maps than on T1- and T2-weighted images (Abduljalil et al., 2003; Abosch et al., 2010; Kerl et al., 2012c, 2013; Deistung et al., 2013b). Although quantitative comparison between studies is difficult due to variations in scan protocols, the CNRs of deep-brain structures on T2^*^ and SW images and corresponding maps are generally higher than those of T2- and T1-weighted images (Kerl et al., 2012c, 2013). For the SNR, the same trend can be seen, although T1-weighted images seem to have a higher SNR than SW images (Kerl et al., 2012c, 2013).

### Perspectives

The improved visualization of the basal ganglia with ultra-high field MRI discussed here provides good perspectives for clinical practice. The clear delineation of DBS target structures and their possible subdivisions may aid in more accurate targeting, which may reduce negative side effects and shorten surgery duration, or it may even allow surgery under general anesthesia. Furthermore, ultra-high field MRI also shows potential for more accurate diagnosis and monitoring of basal ganglia diseases due to, for example, improved identification of the SN pars compacta and SN pars reticulata, which may in its turn facilitate improved patient specific treatments.

In addition, ultra-high field MRI promises to be a versatile tool in clinically oriented research of the deep brain nuclei. It might help us to improve our current understanding of the functionality of the healthy basal ganglia and its disease processes with high resolution functional MRI and connectivity analyses.

### Recommendations

When in the end considering the optimal scan protocol for visualizing the DBS targets for clinical purposes at ultra-high field, both image quality and practical requirements need to be taken into account. In terms of hardware, it is recommended to use a head coil with a high number of receive channels (i.e., 16 or higher). This has been shown to improve the SNR (de Zwart et al., 2004; Wiggins et al., 2006) which is also reflected from the studies described in Table 3. In terms of scan protocol, based on the described literature, we recommend to use a 3D multi-echo GE sequence with an isotropic resolution of 0.5 mm^3^ and partial brain coverage. The 3D sequence facilitates small and isotropic voxel sizes, which ensures good resolution in every plane which is important for distinguishing the STN from the SN. From the multi-echo GE scan, both T2^*^-weighted and susceptibility weighted images as well as T2^*^-maps, R2^*^-maps, and susceptibility maps can be computed, which were shown in the reviewed literature to display best basal ganglia visibility. Since the basal ganglia are located within the same axial oblique slab of approximately 4–5 cm thickness, we advise to shorten scan time by covering only this part of the brain. If more time reduction is required, partial Fourier imaging, elliptical k-space coverage, or parallel imaging can be considered as well.

**Figure 4:**
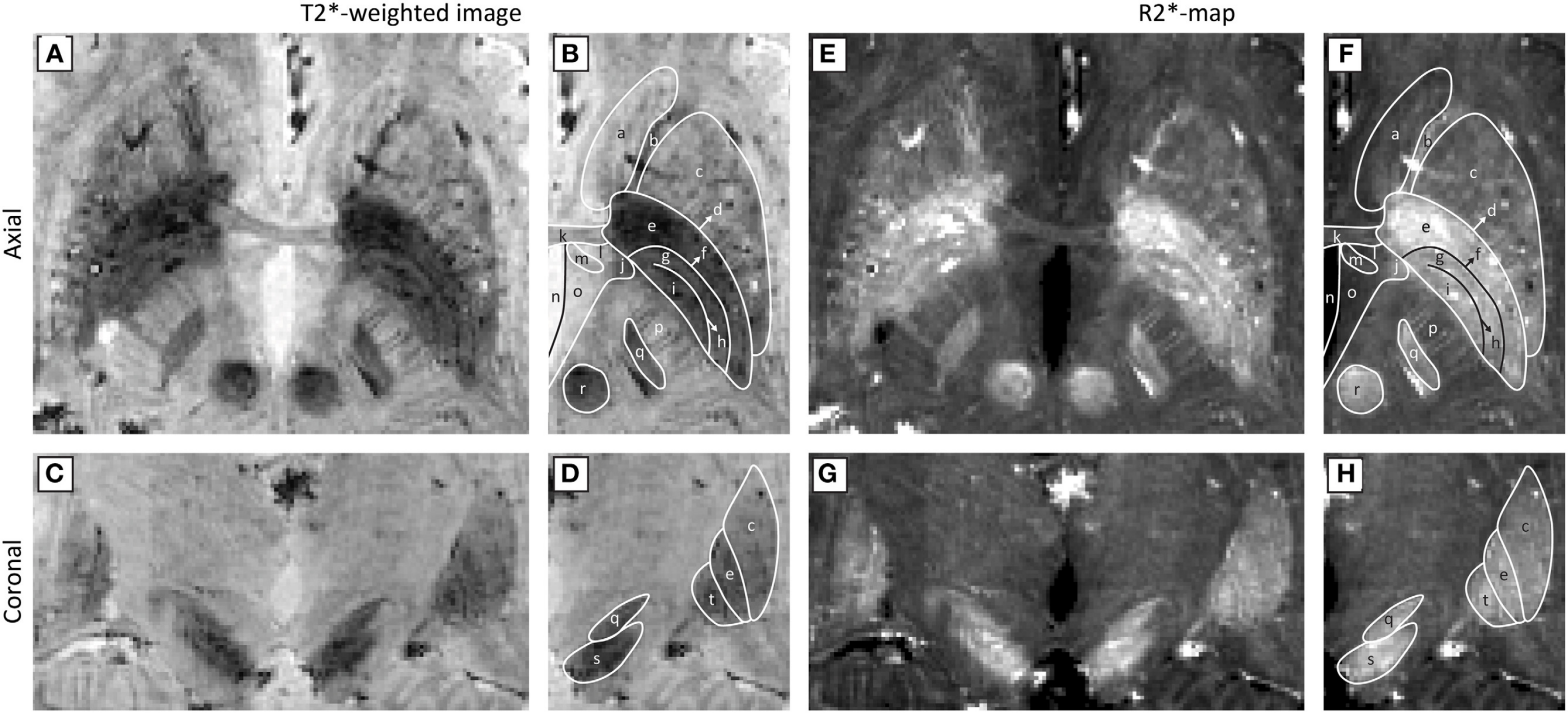
Ultra-high field (7T) axial (A,B,E,F) and coronal (C,D,G,H) T2^*^-weighted images (A–D) and R2^*^-maps (E–H). Panels **(B,D,F,H)** show the anatomical structures that can be identified with the Schaltenbrand and Wahren atlas (Schaltenbrand and Wahren, 2005): (a) caudate nucleus, (b) anterior limb of internal capsule, (c) putamen, (d) lamina pallidi lateralis, (e) external globus pallidus, (f) lamina pallidi medialis, (g) pallidum mediale externum, (h) lamina pallidi incompleta, (i) pallidum mediale internum, (j) inferior thalamic peduncle, (k) anterior commissure, (l) prothalamus, (m) fornix, (n) third ventricle, (o) hypothalamus, (p) posterior limb of internal capsule, (q) subthalamic nucleus, (r) red nucleus, (s) substantia nigra, (t) internal globus pallidus. (Courtesy D. Ivanov).

To support these guidelines, Figure 4 shows an example of a T2^*^-weighted image and an R2^*^-map created with these recommendations. The images were obtained by scanning a healthy volunteer on a 7T MR scanner (Magnetom 7T, Siemens, Erlangen, Germany) at Scannexus (Maastricht, The Netherlands) using a 32-channel phased-array coil (Nova Medical, Wilmington, United States) with a multi-echo 3D GE sequence. Scan time was reduced to 12 min and 23 s by partial brain coverage and 75% partial Fourier imaging (other scanning parameters can be found in Table 3–line 47:48). On these 0.5 mm^3^ isotropic resolution images, the STN can be distinguished from the SN in the coronal plane, and the three laminae of the GP can be identified.

When planning a DBS surgery, the MR images are often registered to CT images, resulting in images that both display the stereotactic frame from the CT image as well as contrast within the brain. This registration may be more reliable, however if a whole brain MR image is available as an intermediate step. Abosch et al. (2010) showed that it is already possible to perform 1 mm^3^ whole brain T1-weighted imaging in 3.5 min, which may be a good candidate for coregistration.

### Limitations

Despite these promising results concerning accurate and high-resolution visualization of the small deep brain (sub)structures, several issues still need to be addressed before they can routinely be employed in direct targeting for DBS.

Firstly, ultra-high field images have an increased risk of geometrical distortions compared to 1.5T images. The severity of these distortions at 7T in deep-brain regions has been investigated in several studies. One study compared the coordinates of marker points in a phantom imaged with 1.5T and 7T MRI to their locations on computed tomography (CT) images (Dammann et al., 2011). The maximum distortion in either x-, y-, or z-direction at 7T was 1.6 mm, which was slightly larger than at 1.5T (0.9 mm). Furthermore, the fewest distortions were observed in the center of the phantom. In another study the distortions in an anthropomorphic phantom between T2^*^-weighted 7T MR and CT images were investigated, revealing a maximum deviation of 0.78 mm (Cho et al., 2010). Finally, registration of 7T T1- and T2-weighted images of the midbrain of PD patients to 1.5T T1- and T2-weighted images showed that mainly rigid body transformations were required and that scaling and skew deformations were small (Duchin et al., 2012). Furthermore, the midbrain region, containing many DBS targets, required the least correction. Quantitative comparison showed that the distances of the T2-weighted images were significantly less than 1 mm suggesting that affine registration of T1- and T2-weighted 7T images to CT images can already provide MR images with midbrain distortions comparable to those of 1.5T images. These few studies suggest that at 7T images can be acquired with distortions smaller than 1 mm in the deep-brain areas.

Secondly, some of the mentioned imaging techniques pose additional challenges in the clinical context. Most studies were performed on young and healthy volunteers. In patients, movement during image acquisition can be less controlled, counteracting the gain in SNR and spatial specificity obtained with ultra-high field. However, newer techniques, such as prospective motion correction might remedy this problem (Maclaren et al., 2013). This approach monitors movement in the scanner with high accuracy and corrects the new image acquisition adaptively according to the new head position. That is, even with large head movements—as observed in many patients—the resulting images are already coregistered and movement artifact free.

In addition, the availability of ultra-high field MR scanners is currently limited. Firstly, the number of scanners that have been installed in the world is limited itself (see Table 2), which is inherent to its high cost in purchase and in operation. Secondly, due to the novel status of ultra-high field MRI, safety precautions regarding metallic objects are often more strict than on 3T systems and the use of ultra-high field MRI is currently only allowed for research purposes.

Finally, direct targeting in DBS suffers from brain shift, intra-operative deformation of the brain compared to preoperative MR images due to difference in head position and cerebrospinal fluid loss. Without compensation for this, it will eventually still limit targeting accuracy. However, this effect is independent of the magnetic field strength and even the pre-operative imaging modality.

## Conclusion

Ultra-high field MRI can reliably and accurately display subdivisions within the basal ganglia and related structures, which especially benefits from T2^*^- and phase-related contrasts. If the limitations concerning image distortions and the availability of the scanners are solved, these technical advances have the potential to improve accuracy of targeting in DBS surgery and the clinical outcome.

### Conflict of interest statement

The authors declare that the research was conducted in the absence of any commercial or financial relationships that could be construed as a potential conflict of interest.

## Abbreviations

CNR: contrast-to-noise ratio
DBS: deep brain stimulation
DWI: diffusion-weighted imaging
FLAIR: fluid attenuated inversion recovery
FLASH: fast low-angle shot
GE: gradient echo
GP: globus pallidus
GPe: external globus pallidus
GPi: internal globus pallidus
GRASE: gradient and spin-echo
MIP: minimum intensity projection
MPRAGE: magnetization-prepared rapid acquisition of gradient echo
PD: Parkinson's disease
PIAF: parallel imaging acceleration factor
RN: red nucleus
rZI: rostral part of ZI
SE: spin-echo
SN: substantia nigra
SNR: signal-to-noise ratio
SPACE: sampling perfection with application of optimized contrasts using different flip angle evolutions
SW: susceptibility-weighted
SWI: susceptibility-weighted imaging
TDI: track-density imaging
TE: echo time
TR: repetition time
TSE: turbo spin-echo
ZI: zona incerta.
